# Phylogenetic analyses of *Ixodes rugicollis* with notes on its morphology in comparison with *Ixodes cornutus*

**DOI:** 10.1186/s13071-023-05718-z

**Published:** 2023-03-16

**Authors:** Sándor Hornok, Andrei D. Mihalca, Jenő Kontschán, Nóra Takács, Denis Fedorov, Olivier Plantard, Attila D. Sándor

**Affiliations:** 1 Department of Parasitology and Zoology, University of Veterinary Medicine, Budapest, Hungary; 2 ELKH-ÁTE Climate Change: New Blood-Sucking Parasites and Vector-Borne Pathogens Research Group, Budapest, Hungary; 3 Department of Parasitology and Parasitic Diseases, University of Agricultural Sciences and Veterinary Medicine, Cluj-Napoca, Romania; 4 Plant Protection Institute, Centre for Agricultural Research, Budapest, Hungary; 5 Department of Plant Sciences, Albert Kázmér Faculty of Mosonmagyaróvár, Széchenyi István University, Mosonmagyaróvár, Hungary; 6 Zoological Institute of the Russian Academy of Sciences (ZIN-RAS), St. Petersburg, Russia; 7 Oniris, INRAE, BIOEPAR, Nantes, France

**Keywords:** *Ixodes*, Carnivores, Mustelidae, cox1, 16S rRNA gene, 18S rRNA gene, 28S rRNA gene

## Abstract

**Background:**

The subgenus *Pholeoixodes* contains *Ixodes* species typically associated with birds that nest in cavities or with carnivorous mammals that are burrow-dwelling. Among ticks infesting the latter, *Ixodes rugicollis* is regarded as the rarest species in the western Palearctic. Despite the unique morphology of this species, its identification (especially of subadult stages) is difficult, and molecular-phylogenetic data to offer other diagnostic methods and a better understanding of its taxonomy are not available.

**Methods:**

In this study, a female and a male of *I. rugicollis* were collected in Romania. The female was compared morphologically to another female of this species collected in France and to the lectotype of *Ixodes cornutus* (from Tajikistan), which has similar morphology and host association. Following DNA extraction, two mitochondrial (cytochrome *c* oxidase subunit I: *cox1* and the 16S rRNA gene) and two nuclear genetic markers (18S and 28S rRNA genes) of *I. rugicollis* were amplified and analyzed in a phylogenetic context.

**Results:**

Females of *I. rugicollis* and *I. cornutus* differed in the shape of their palps, scutum and areae porosae and the size of peritremes, but they were similar in palpal setal length, dental formula and arrangement of anal setae. Measurements of two *I. rugicollis* females examined were not less different from each other than from *I. cornutus*. Phylogenetically, *I. rugicollis* clustered with other members of its subgenus. The topology of all trees showed the position of bat-associated tick species of the subgenus *Eschatocephalus* among *Pholeoixodes* species.

**Conclusions:**

For the first time to our knowledge, this study provides high-resolution digital pictures of male and female *I. rugicollis* as well as corresponding molecular data. Morphological comparison of this species with *I. cornutus* could not resolve uncertainties in the validity of the latter species, which can only be accomplished after collecting new specimens of *I. cornutus* and consequent molecular comparisons. This study includes the first comprehensive molecular-phylogenetic analysis of western Palearctic *Pholeoixodes* species based on both nuclear and mitochondrial genetic markers and including *I. rugicollis*. The results of these confirm the phylogenetic position of subgenus *Eschatocephalus* within *Pholeoixodes*, justifying the need to merge them to comply with the taxonomic criterion of monophyly.

**Graphical Abstract:**

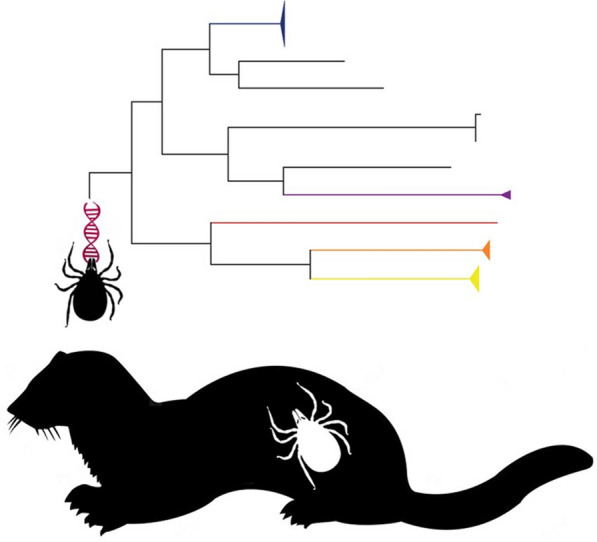

**Supplementary Information:**

The online version contains supplementary material available at 10.1186/s13071-023-05718-z.

## Background

Among hard ticks (Acari: Ixodidae), the genus *Ixodes* Latreille, 1795, contains the highest number of valid species [1], and as many as eight of its subgenera occur in the western Palearctic [2]. The subgenus *Pholeoixodes* Schulze, 1942, was established based on common morphological features of its members [3], as exemplified by the relatively short palps, absence of auriculae and subapical dorsal hump on the tarsi [4, 5]. *Pholeoixodes* species also share ecological traits as they are usually associated with burrow-dwelling mammals and terrestrial birds that nest in cavities (tree holes or burrows). Species of this subgenus infesting mammals, particularly carnivores (mainly Canidae, Mustelidae) and hedgehogs (Erinaceidae), in the western Palearctic include *Ixodes canisuga* Johnston, 1849, *I. kaiseri* Arthur, 1957, *I. crenulatus* Koch, 1844, *I. hexagonus* Leach, 1815, and *I. rugicollis* Schulze & Schlottke, 1929. Although during the past few years morphological keys were published to differentiate adults and developmental stages of this subgenus [6, 7], identification of western Palearctic *Pholeoixodes* species remains a difficult task for even experienced taxonomists.

This is well reflected by diagnostic uncertainties related to the most scarcely collected and least studied species of this subgenus in Europe, i.e. *I. rugicollis*. This species was reported from stone martens (*Martes foina*) and red foxes (*Vulpes vulpes*) in France [8], pine martens (*Martes martes*) in Germany [9], dogs and cats in Poland [10], stone martens (*M. foina*) in Switzerland [11] and Austria [12] and European polecat (*Mustela putorius*) in Romania [13]. Historically, even *I. rugicollis* adults were problematic to recognize, because in the original illustration [9] and later drawings [14], the characteristic frontal projections are not shown, and its proposed synonymy with *I. cornutus* Lotozky, 1956 [15], also led to misidentification [16]. Regarding developmental stages, when *I. rugicollis* was reported and probably correctly identified in Austria [12], the criteria of morphological recognition were not mentioned, and the picture provided by the authors does not show the most important diagnostic feature of nymphs, i.e. that the sides of basis capituli are parallel [8, 17]. These difficulties could be prevented if molecular identification of *I. rugicollis* were to become possible, but until this study there has been no sequence of this species available in GenBank.

The phylogenetic relationships of western Palearctic *Pholeoixodes* species were also reported, and it was shown that this subgenus is only monophyletic if containing bat-associated ticks of the subgenus *Eschatocephalus* [6]. However, these results were based exclusively on mitochondrial markers and did not include the rare species *I. rugicollis*. Therefore, considering the above, the aims of the present study were threefold: (i) to obtain a barcoding sequence of *I. rugicollis* and thus to prevent further difficulties in its morphological identification, especially in case of subadult stages; (ii) to provide and to evaluate the most complex phylogeny of western Palearctic *Pholeoixodes* ticks based on two mitochondrial and two nuclear markers, now including *I. rugicollis*; (iii) to investigate the morphology of the latter species compared with *I. cornutus*.

## Methods

### Sample collection and morphological identification

The most important sample that served as the initiative for this study, *I. rugicollis* (mating female and male), were collected in Fersig, Romania, from a stone (or beech) marten (*M. foina*) in January 2018. In addition, measurements of an *I. rugicollis* female from the collection of P. C. Morel were also performed (removed from *M. foina* in Dommartin, France, on March 27, 1973). Further *Pholeoixodes* ticks used for DNA extraction and *cox1* (cytochrome *c* oxidase subunit I), 16S, 18S and 28S rRNA gene PCR analyses were as follows: *Ixodes arboricola* (nymph collected from *Parus major* in Ócsa, Hungary) and *I. lividus* (female collected from *Riparia riparia* in Ócsa, Hungary). In the latter analyses two further species were also included: *Ixodes ricinus* (female collected from the vegetation in Sümeg, Hungary) and *I. trianguliceps* (larva collected from *Myodes glareolus* in the Leningrad region, Russia).

All ticks were stored in 96% ethanol, and their species were morphologically identified according to standard keys (*I. rugicollis* female: [8]; *I. rugicollis* male: [18]; other species: [19]). In addition, the morphology of the syntype (female) of *I. rugicollis* was also studied previously by the authors [6], and this was considered here for its morphological identification. Pictures and measurements of *I. rugicollis* were made with a VHX-5000 digital microscope (Keyence Co., Osaka, Japan) while ensuring the appropriate angle of view, i.e. perpendicular to the surface under evaluation. In addition, the type specimen (lectotype) of *I. cornutus* (female collected from *Mustela erminea* in Tajikistan) was studied with an Altami B151060063 binocular microscope (OOO Altami, Russia) and a Levenhuk M 1400 plus digital camera (Levenhuk, Inc., USA) at the Zoological Institute of the Russian Academy of Sciences, St. Petersburg, Russia. The description of this species was translated, and the drawings are taken from Filippova [20] (Additional File 1).

### DNA extraction

Tick surfaces were disinfected with sequential washing in 10% sodium-hypochlorite, tap water and distilled water. DNA was extracted with the QIAamp DNA Mini Kit (QIAGEN, Hilden, Germany) according to the manufacturer's instructions, including an overnight digestion in tissue lysis buffer and Proteinase K at 56 °C. An extraction control (tissue lysis buffer) was also processed in each set of tick samples to monitor cross-contamination. Additional DNA extracts from previous studies used for 18S and 28S rRNA gene PCRs and phylogenetic analyses are as follows: *Ixodes vespertilionis* and *I. ariadnae* (collected from *Rhinolophus ferrumequinum* and cave wall in Pilis Mountains, respectively, in Hungary: [21]) as well as *I. frontalis* and *I. acuminatus* (collected from birds, *Erithacus rubecula* and *Anthus pratensis*, respectively, in Malta: [22]).

### Molecular taxonomic analyses

An approximately 710-bp-long fragment of the *cox1* gene was amplified with a conventional PCR modified from Folmer et al. [23]. The primers HCO2198 (5ʹ-TAA ACT TCA GGG TGA CCA AAA AAT CA-3′) and LCO1490 (5′-GGT CAA CAA ATC ATA AAG ATA TTG G-3′) were used in a reaction volume of 25 µl, containing 1 U (0.2 µl) HotStarTaq Plus DNA polymerase, 2.5 µl 10 × CoralLoad Reaction buffer (including 15 mM MgCl_2_), 0.5 µl PCR nucleotide mix (0.2 mM each), 0.5 µl (1 µM final concentration) of each primer, 15.8 µl ddH_2_O and 5 µl template DNA. During the amplification, the initial denaturation step at 95 °C for 5 min was followed by 40 cycles of denaturation at 94 °C for 40 s, annealing at 48 °C for 1 min and extension at 72 °C for 1 min. Final extension was performed at 72 °C for 10 min.

Another PCR was used to amplify an approximately 460-bp fragment of the 16S rDNA gene of Ixodidae [24], with the primers 16S + 1 (5′-CTG CTC AAT GAT TTT TTA AAT TGC TGT GG-3ʹ) and 16S-1 (5ʹ-CCG GTC TGA ACT CAG ATC AAG T-3′). Reaction components and cycling conditions were the same as above, except for annealing at 51 °C. In addition, two nuclear genetic markers were also amplified: an approximately 1700-bp-long fragment of the 18S rRNA gene with the primers NS1 (5′-GTA GTC ATA TGC TTG TCT C-3′) and NS4a (5′-GCC CTT CCG TCA ATT CCT TTA AG-3′) [25] as well as an approximately 700-bp-long fragment of the 28S rRNA gene with the primers 28ScF (5′-GTG GTA GCC AAA TGC CTC GTC ATC-3′) and 28SR (5′-GAA TTC TGC TTC ACA ATG ATA GGA AGA GCC-3′) as reported [26].

### PCR controls, sequencing and phylogenetic analyses

In all PCRs, non-template reaction mixture served as negative control. Extraction and negative controls remained PCR negative in all tests. Purification and sequencing of the PCR products were done by Biomi Ltd. (Gödöllő, Hungary). Quality control and trimming of sequences were performed with the BioEdit program. Obtained sequences were compared to GenBank data by the nucleotide BLASTN program (https://blast.ncbi.nlm.nih.gov). New sequences were submitted to GenBank (*cox1* gene: OP997945 for *I. rugicollis* and OP997946 for *I. arboricola*, 16S rRNA gene: OP998019 for *I. rugicollis*, 18S rRNA gene: OP998033-OP998044, 28S rRNA gene: OP998050-OP998063). Sequences from other studies (retrieved from GenBank) included in the phylogenetic analyses had nearly 100% coverage with sequences from this study. In the *cox1* and 16S rRNA gene phylogenetic analyses, unrooted trees were made for evaluating interspecific relationships. For this purpose, all sequences of *Ixodes canisuga*, *I. kaiseri* and *I. hexagonus* were used from our previous study [6]. In the phylogenetic analyses of nuclear markers (18S and 28S rRNA genes) *Ixodes* (*Ceratixodes*) *uriae* and *I.* (*Exopalpiger*) *trianguliceps* were used as outgroups. Sequence datasets were resampled 1000 times to generate bootstrap values. Initial phylogenetic analyses were conducted with the neighbor-joining method using p-distances and maximum likelihood method with Jukes-Cantor model by the MEGA version 7.0 program [27].

## Results

### Morphology of *I. rugicollis* and its comparison with *I. cornutus*

**Fig. 1:**
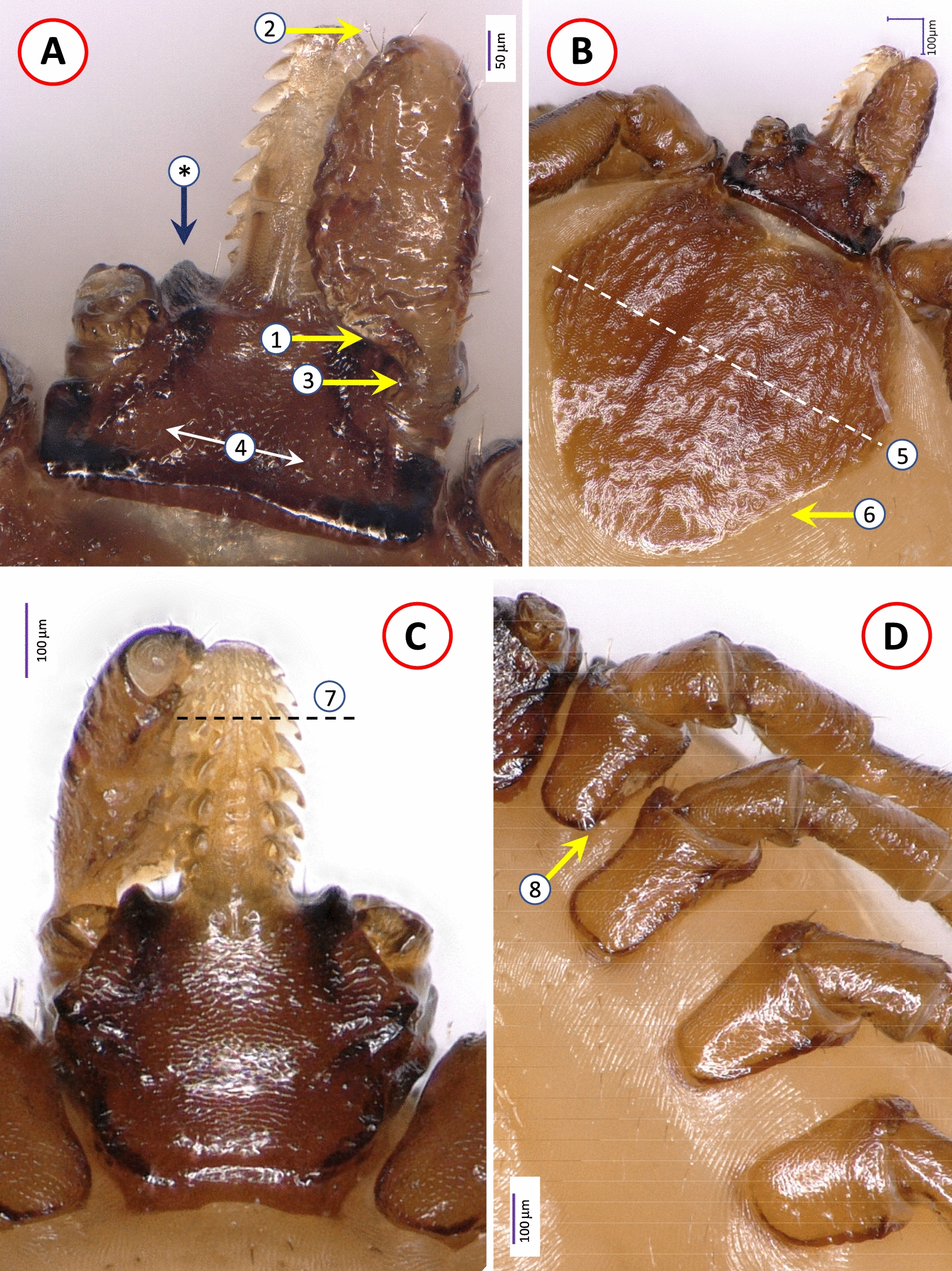
Key morphological characters of *Ixodes rugicollis* female, dry mounting: **A** dorsal view of basis capituli, hypostome and palp (asterisk and blue arrow mark the frontal protuberance); **B** dorsal view of scutum and basis capituli (dashed line marks maximum width of the scutum); **C** ventral view of basis capituli, hypostome and palp (dashed line separates the apical quarter of hypostome with dental formula higher than 2/2); **D** coxae. Numbers 1–8 mark structures of diagnostic importance described in Table 1

**Fig. 2:**
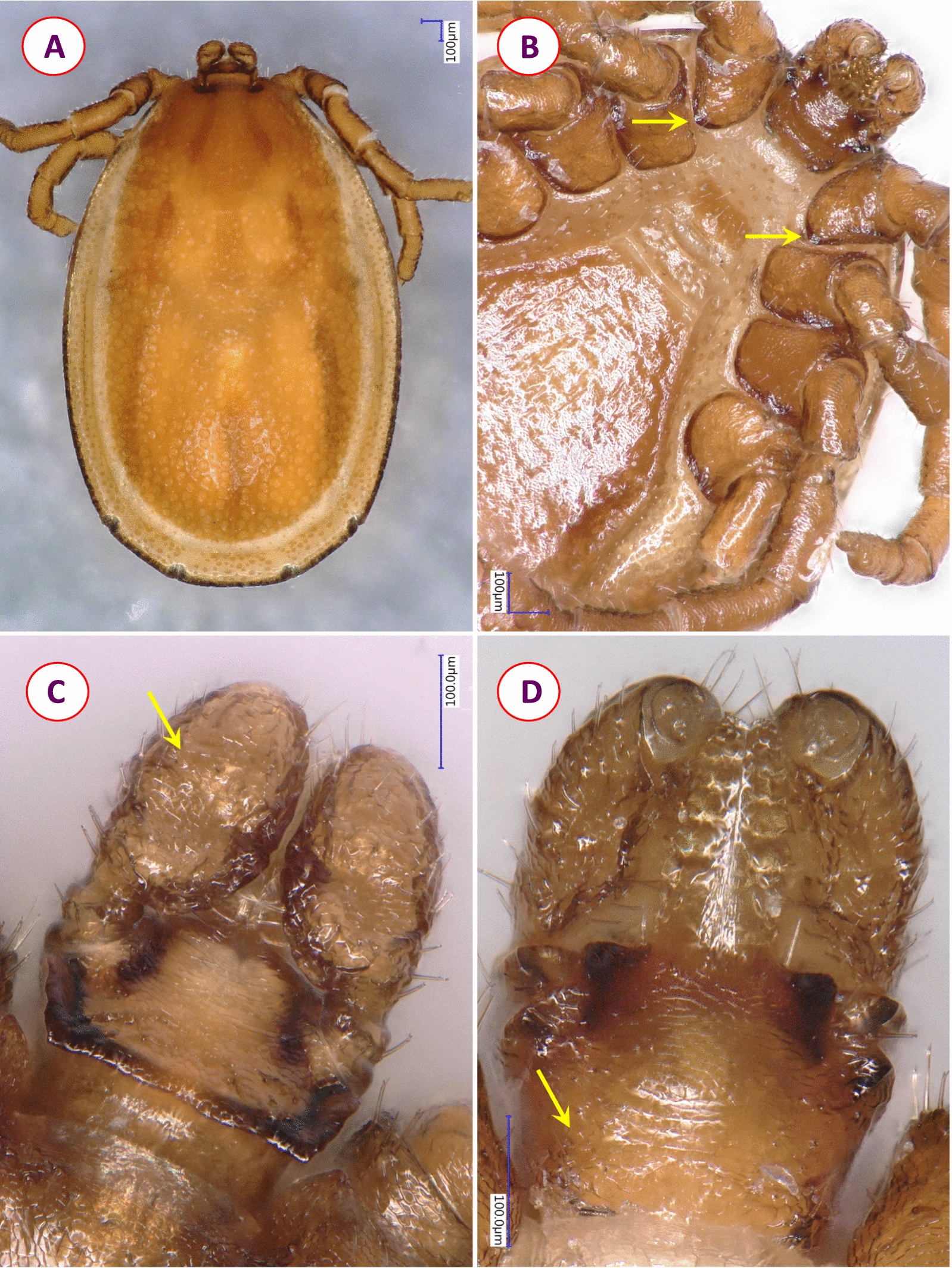
Key morphological characters of *Ixodes rugicollis* male, **A** wet and **B**–**D** dry mounting: **A** habitus, dorsal view; **B** ventral view of coxae (yellow arrows mark short internal spur on coxae I); **C** dorsal view of basis capituli and palps (yellow arrow indicates wrinkled surface of palp); **D** ventral view of basis capituli, hypostome and palps (yellow arrow indicates short hairs behind the ridge replacing auriculae)

The female tick from *M. foina* was morphologically identified as *I. rugicollis* according to the frontal projections and small, well-separated porose areas on the basis capituli as well as the uniformly wrinkled (rugose) surface of the scutum and palps, the latter with a narrow “stalk” (Fig. 1). The male mating with this female was also identified as *I. rugicollis* based on the wrinkled surface of palps, small external spur on each coxa and the presence of short setae below the auricular ridge ventrally on the basis capituli (Fig. 2).

The female of *I. rugicollis* and the lectotype of *I. cornutus* were also compared morphologically. In several characters relevant to recognize the subgenus *Pholeoixodes* in general, and its two members in particular, they were different (as exemplified by the shape of the palps, scutum and areae porosae), but at the same time other diagnostic characters, such as the shortness of palpal setae, dental formula and arrangement of anal setae, were similar for both (Table 1; Additional file 2, 3). Regarding measurements of the two *I. rugicollis* females and the lectotype of *I. cornutus*, the length, width and index (length-to-width ratio) of the scutum, basis capituli and palps anterior to their “stalk” showed minor differences between the two species, but also between the two conspecific females (Additional file 4). On the other hand, the peritremes of *I. rugicollis* females were < 200 μm (Additional file 3B), in contrast to those of *I. cornutus* measuring nearly 300 μm (Additional file 1).

**Table 1:** Summary of most important diagnostic characters that differ or are similar between *Ixodes rugicollis* and *I. cornutus* females

Character	*Ixodes rugicollis* (illustration)	*Ixodes cornutus* (illustration)
Palps dorsally	1.Medially strongly curved in its posterior half, segment II. posteriorly tapering at nearly perpendicular angle to “stalk” (Fig. 1A) 2.All palpal setae short (< 30 μm)	1.Medially equally curved in its anterior and posterior halves, segment II. posteriorly tapering at acute angle to “stalk” (Additional File 2A) 2.All palpal setae short (< 30 μm) (Additional File 1)
Width of “stalk” of palpal segment II	3.Approx. one third of palpal width (Fig. 1A)	3.Approx. half of palpal width (Additional File 2A)
Areae porosae	4.Small, circular, with approx. 130 μm distance between them (Fig. 1A)	4.Irregularly oval, with approx. 50 μm distance between them (Additional File 2A)
Scutum shape	5.Slightly broader than long, broadest at approx. mid-length, 6.Posterolateral margin concave (Fig. 1.B, Additional File 3A)	5.Slightly longer than broad, broadest anteriorly at approx. one third of length, 6.Posterolateral margin straight (Additional File 2.A)
Hypostome dentition	7.2/2, apically (anterior quarter) 3/3 or higher (Fig. 1C)	7.2/2, apically (anterior quarter) 3/3 or higher (Additional File 1)
Coxa I	8.Short internal spur present (Fig. 1D)	8.Short internal spur inapparent (Additional File 2B)
Anal valves	9.Anterior three setae vertically, posterior two setae horizontally (Additional File 3C)	9.Anterior three setae vertically, posterior two setae horizontally (Additional File 1)

Serial numbers correspond to numbers in white circles of figures referred to in the table. For *I. cornutus* see also the drawings by Filippova (1977) in Additional File 1

### Molecular-phylogenetic relationships of *I. rugicollis* and other western Palearctic *Pholeoixodes* species

The amplified part of the *cox1* gene was identical between the female and male of *I. rugicollis*, showing the highest but only 85.6% (482/563 bp) sequence identity to that of *I. vespertilionis* reported from France (KR902757). Among Palearctic *Pholeoixodes* species, the 16S rRNA gene of *I. rugicollis* had the highest sequence identity, 89.1% (361/405 bp), to *I. hexagonus* from Croatia available in GenBank (KY962076). The 18S rRNA sequence of *I. rugicollis* differed from that of *I. hexagonus* (JN018307) in seven positions but only in six positions when compared to *I. ricinus* from France (GU074648) (meaning 1026/1033 or 1027/1033 bp, i.e. 99.3 or 99.4% identities, respectively). The 28S rRNA sequence of *I. rugicollis* was most similar to that of *I. hexagonus* (JN018404: 599/600 bp = 99.8% identity), followed by *I. simplex* (KY457498: 596/600 bp = 99.3% identity).

**Fig. 3:**
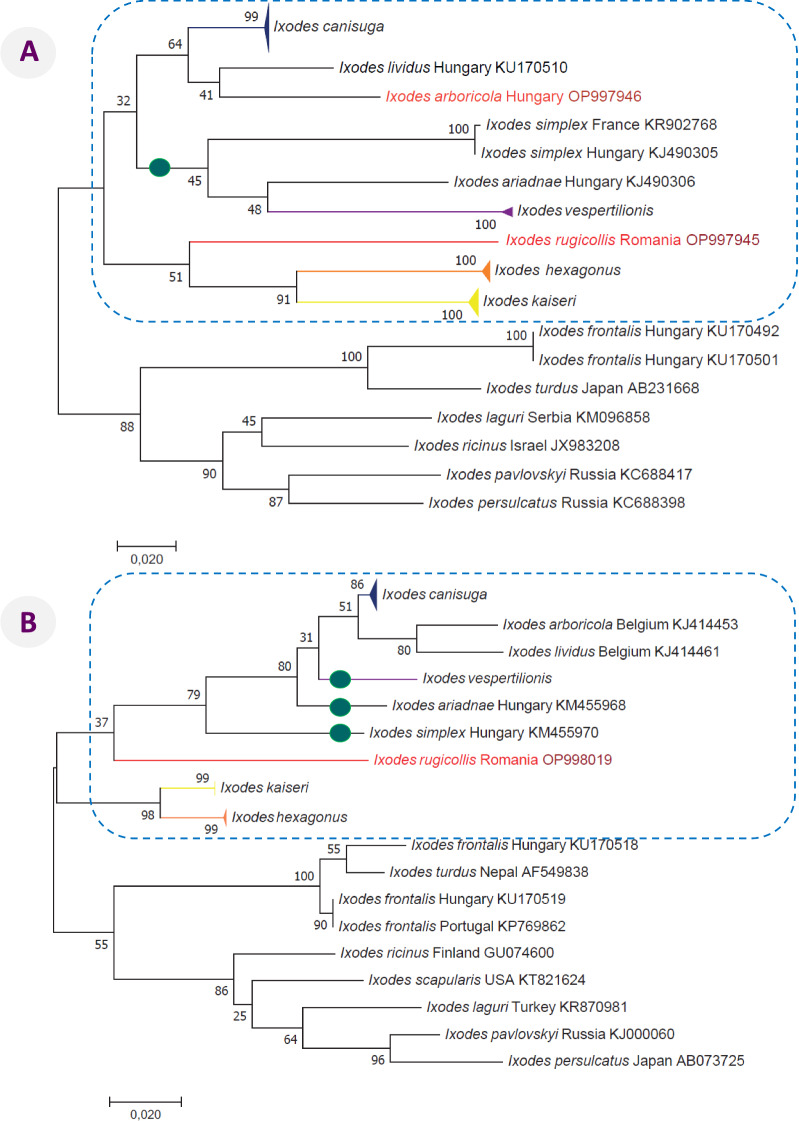
Phylogenetic tree of *Ixodes* species based on **A** *cox1* and **B** 16S rRNA gene sequences. In each row of individual sequences, the country of origin and GenBank accession number are shown after the species name. For *Pholeoixodes* species of carnivores and *I. vespertilionis*, all sequences from [6], were included, and their branches are shown collapsed with separate color and triangle at the end. *Ixodes rugicollis* is marked with a red branch and all sequences from this study with red fonts and maroon accession numbers. The subgenus *Pholeoixodes* is surrounded by blue dashed line, whereas *Eschatocephalus* species are marked with a green dot on their branch. The evolutionary history was inferred by using the maximum likelihood method based on the Jukes-Cantor model. The tree with the highest log likelihood is shown. The percentage of trees in which the associated taxa clustered together is shown next to the branches. Initial tree(s) for the heuristic search were obtained automatically by applying neighbor-join and BioNJ algorithms to a matrix of pairwise distances estimated using the maximum composite likelihood (MCL) approach and then selecting the topology with superior log likelihood value. The tree is drawn to scale, with branch lengths measured in the number of substitutions per site. The analysis involved 59 and 43 nucleotide sequences for the *cox1* and 16S rRNA genes, and there were a total of 564 and 363 positions in the final dataset, respectively. All positions containing gaps and missing data were eliminated

In the phylogenetic analyses of both mitochondrial (Fig. 3) and nuclear markers (Additional file 5), *I. rugicollis* clustered with other representatives of the subgenus *Pholeoixodes*. In the *cox1* analysis *I. rugicollis* was a sister species to *I. hexagonus* and *I. kaiseri* (Fig. 3A), in the 28S rRNA tree to *I. simplex* (Additional file 5B), but in none of the four phylogenetic trees to *I. canisuga*. The topology of all trees showed the position of bat-associated tick species of the subgenus *Eschatocephalus* among *Pholeoixodes* species. Interestingly, in the 16S rRNA analysis, the subgenus *Eschatocephalus* was paraphyletic (Fig. 3B), and in the 28S rRNA phylogenetic tree, although with low support, it was also not monophyletic (Additional file 5B). In the 18S rRNA phylogenetic analysis, *I. rugicollis* belonged to a clade including *Trichotoixodes* species and members of the *I. ricinus* complex (Additional file 5A) and this was relatively well supported (86%).

## Discussion

This study provides high-resolution digital pictures of *I. rugicollis* and corresponding molecular data. The importance of this can only be assessed when considering that *I. rugicollis* is the most scarcely collected and least studied western Palearctic *Pholeoixodes* species. Males of *I. rugicollis* are especially rare, missing from the collections listed in the redescription of female and developmental stages of this species from France and other countries [8]. Accordingly, the male was described elsewhere [18]. This study also contains, for the first time to our knowledge, high-resolution pictures of the male of *I. rugicollis* and thus can hopefully aid the appropriate recognition of this sex in future studies.

The frontal projections of the basis capituli are regarded as the most important characters of *I. rugicollis* females. However, the capitulum of another western Palearctic species of its subgenus, *I. canisuga*, may also resemble this character, because margins of the flattened, plateau-like frons around the hypostomal base can be elevated [16]. In addition, the wrinkling of the lateral fields of the scutum was also reported in *I. canisuga* [6], confirming the need for molecular barcoding of *I. rugicollis* for which the present study provided sequences of four genetic markers. This is even more desirable for the identification of subadult stages to prevent uncertainties in their reports [12].

*Ixodes rugicollis* is known to occur exclusively in Europe. However, the presence of frontal projections on the basis capituli of females of *I. rugicollis* is shared with *I. cornutus* reported from central Asia. The description and illustration of *I. cornutus* are based on a female lectotype collected from *Mustela erminea* in Tajikistan ([20]). Although the synonymy of *I. rugicollis* and *I. cornutus* was proposed [15], most taxonomical sources maintained the status of the latter species as provisionally valid [1, 28, 29]. Here, for the first time to our knowledge, the morphology of these two species was compared in detail. Based on the results, *I. rugicollis* and *I. cornutus* share several diagnostically important features, including the frontal projections, wrinkled (rugose) surface of the scutum and palps, hypostome dentition and arrangement of anal valve setae. Although the latter character is frequently neglected in the differential diagnosis of ixodid species, it was shown to be different between *I. rugicollis* and the closely related species *I. crenulatus* [14].

At the same time, other characters are apparently different between these two species, as exemplified by the shape of palps (changing if not perpendicularly viewed), areae porose (which are difficult to see because of the high degree of sclerotization) and scutal index. However, the latter two characters are strongly influenced by (1) individual variation, as shown here between two *I. rugicollis* females, (2) the existence of different morphotypes within the same *Pholeoixodes* species [6] and (3) the angle of view (sometimes difficult to assess) and (4) even the state of engorgement in case of the scutal index [30]. Unfortunately, no better quality pictures of *I. cornutus* lectotype could be made, and its further morphological examination is currently not possible. These uncertainties do not allow to draw a final conclusion on the validity of *I. cornutus* as a separate species, and a molecular comparison with *I. rugicollis* will be inevitable in the future when access to suitable, recently collected material for such investigation is possible.

The existence of two morphologically similar tick species with overlapping host spectra (i.e. Mustelidae for both *I. rugicollis* and *I. cornutus*) probably cannot be explained by association with different host subfamilies (Martinae vs. Mustelinae, respectively), particularly because *I. rugicollis* was also reported from Canidae, Felidae and Mustelinae. Another member of its subgenus (*Pholeoixodes*), the fox tick (*I. kaiseri*), was reported to be genetically very similar between distant continental regions of central Europe and central Asia [31], probably because fox populations are confluent between these regions [32]. The only known host of *I. cornutus*, the stoat (*M. erminea*), has a broad geographical range in Eurasia. Although they are sympatric with other mustelids, such as the common weasel (*M. nivalis*), they typically occupy different habitats (wetlands vs. grasslands and forests: [33]). Therefore, more data on the host spectrum and geographical range of *I. cornutus* are necessary to elucidate whether it occurs in sympatry with *I. rugicollis* and whether their host associations are habitat-dependent.

This study provides the first comprehensive molecular and phylogenetic analyses of western Palearctic *Pholeoixodes* species based on both nuclear and mitochondrial genetic markers and including the rare species *I. rugicollis*. Prior to this study only one 18S and two 28S rRNA sequences of western Palearctic *Pholeoixodes* species were available for comparison in GenBank, and now this is extended to all valid species except *I. crenulatus*. Based on the mitochondrial *cox1* and 16S rRNA genes, it was already proposed that this subgenus is not monophyletic unless including bat-associated tick species of the subgenus *Eschatocephalus* [6]. This was confirmed here using nuclear genetic markers. In addition, it is interesting to note that in none of the four phylogenetic trees was *I. rugicollis* a sister species to *I. canisuga*, which appears to be the most closely related *Pholeoixodes* species in both its morphology and host range.

Although both mitochondrial and nuclear markers chosen in this study for molecular and phylogenetic analyses are well established and widely used genetic markers in tick systematics [25, 34], the relationships within the subgenus *Pholeoixodes* were only supported by low to moderately high bootstrap values. Thus, the results also justify the future need to investigate more resolutive molecular markers, for example the whole mitogenome [35], in combination with nuclear markers.

## Supplementary Information

**Additional file 1: **Description and drawings of *Ixodes cornutus* by Filippova [20].

**Additional file 2: **Low-resolution pictures of *Ixodes cornutus* lectotype (female) stored at ZIN-RAS (St. Petersburg, Russia): (A) dorsal view of scutum and basis capituli in dry mount (dashed line marks maximum width of the scutum); (B) ventral view of anterior idiosoma in wet mount. Numbers between 1 and 8 mark structures of diagnostic importance described in Table 1.

**Additional file 3: **Morphological characters of *Ixodes rugicollis* female: (A) scutum and basis capituli in wet mount shown in a slightly posterior view (the dark arrow indicates concave posterolateral margin of the scutum); (B) respiratory opening and (C) anal valves with five pairs of hair (marginated white circles indicate the base of these hairs to highlight their arrangement).

**Additional file 4: **Measurements of *Ixodes rugicollis* and *I. cornutus*.

**Additional file 5: **Phylogenetic tree of ixodid ticks based on (A) 18S and (B) 28S rRNA gene sequences. In each row of individual sequences, the country of origin and the GenBank accession number are shown after the species name. *Ixodes rugicollis* is marked with red branch (A) and all sequences from this study with red fonts and maroon accession numbers. The subgenus *Pholeoixodes* is surrounded by a blue dashed line, and *Eschatocephalus* species are marked with a green dot on the branch. The evolutionary history was inferred by using the (A) maximum likelihood method based on the Jukes-Cantor model or (B) neighbor-joining method and p-distance model. The tree with the highest log likelihood is shown. The percentage of trees in which the associated taxa clustered together is shown next to the branches. The tree is drawn to scale, with branch lengths measured in the number of substitutions per site. The analysis involved 21 and 17 nucleotide sequences for the 18S and 28S rRNA genes, and there were a total of 1023 and 586 positions in the final dataset, respectively. All positions containing gaps and missing data were eliminated.

## Data Availability

The sequences obtained during this study are deposited in GenBank under the following accession numbers: *cox1* gene: OP997945 for *I. rugicollis* and OP997946 for *I. arboricola*, 16S rRNA gene: OP998019 for *I. rugicollis* and 18S rRNA gene: OP998033-OP998044, 28S rRNA gene: OP998050-OP998063. All other relevant data are included in the manuscript and the references or are available upon request by the corresponding author.

## Abbreviations

ZIN-RAS: Zoological Institute of the Russian Academy of Sciences
cox1: Cytochrome *c* oxidase subunit I
