# Supplementary material for: Phylogenetic analyses of Ixodes rugicollis with notes on its morphology in comparison with Ixodes cornutus
Source: Parasit Vectors. 2023 Mar 16;16:106. doi: 10.1186/s13071-023-05718-z (PMC10022209; doi:10.1186/s13071-023-05718-z)
Supplement: Supplementary file 1 — Additional file 1: Description and drawings of Ixodes cornutus by Filippova [20]. [file 13071_2023_5718_MOESM1_ESM.pdf]

Ixodes cornutus Lotozkyi, 1956

Lotozkyi, 1956: 27 – 29 (female).[1]

Female. The scutum (Fig. ***1***) is narrowed in the posterior part; its length slightly exceeds its width (1.26 x 1.19 mm). The scapulae look like equilateral triangles. The lateral grooves are not manifested. The cervical-lateral fields bear several longitudinal ridges along their entire lenght. The cervical grooves have the shape of longitudinal pits in the middle part of the scutum. The punctation is very large and immersed, especially in the posterior part of the median and cervical-lateral field, between the ridges. The spiracular plate is slightly elongated in the transverse direction (0.28 x 0.29 mm), the ostium is located almost in the center. Both halves of the anal valve are with 5 – 5 setae (Fig. ***2***).

The posterior margin of the basis capituli on its dorsal side (Fig. ***3***) has a barely noticeable medial groove, the cornua are absent. The porose area is immersed and has an irregularly oval shape. There are the tooth-like projections on the anterior part of the basis capituli, lateral to the cheliceral sheath. Therefore, the ventral side of the basis capituli is peculiarly shaped due to these anterior projections. They are triangular in longitudinal section (Figs. ***3***, ***4***). The posthypostomal setae are displaced in comparison to the norm for the subgenus *Pholeoixodes* and are located as shown in Fig. ***4***. The auriculae are irregularly arcuate, their margins are indistinctly wavy. Dorsally, the palps look as shown in Fig. ***3***. The hypostome has 4–4 longitudinal rows of the teeth in its apical part and 2–2 rows along its entire length. The apical teeth are small (the apex of the hypostome is slightly damaged in the lectotype).

All the coxae (Fig. ***5***) are without spurs. The tarsus 1 is typical of the subgenus (Fig. ***6***); 0.52 mm long.

The male, nymph, larva – unknown.

Distribution and ecology. The species was described from 2 identical females (Lotozkyi, 1956) that were found in Tajikistan in the eastern part of Peter the First Range, by the source of the Divansu river (the basin of the Surkhob river), near the Oshanin glacier*, on a stoat – *Mustela erminea* L. * 4.07.1954.

---

[1] Lectotype: Tajikistan, Peter the First Range, the source of the Divansu river, 4.07.1954, from the stoat – Mustela erminea L., det. B. V. Lotozkyi. Stored at the Zoological institute of the Russian Academy of Sciences: И – 845 -1970.

Figure: *Ixodes cornutus* Lotozkyi. Female (Lectotype).

1 – scutum, *2* – anal valve, *3* – dorsal side of the gnathosoma, *4* – ventral side of the gnathosoma, *5* – coxae, *6* – tarsus 1.

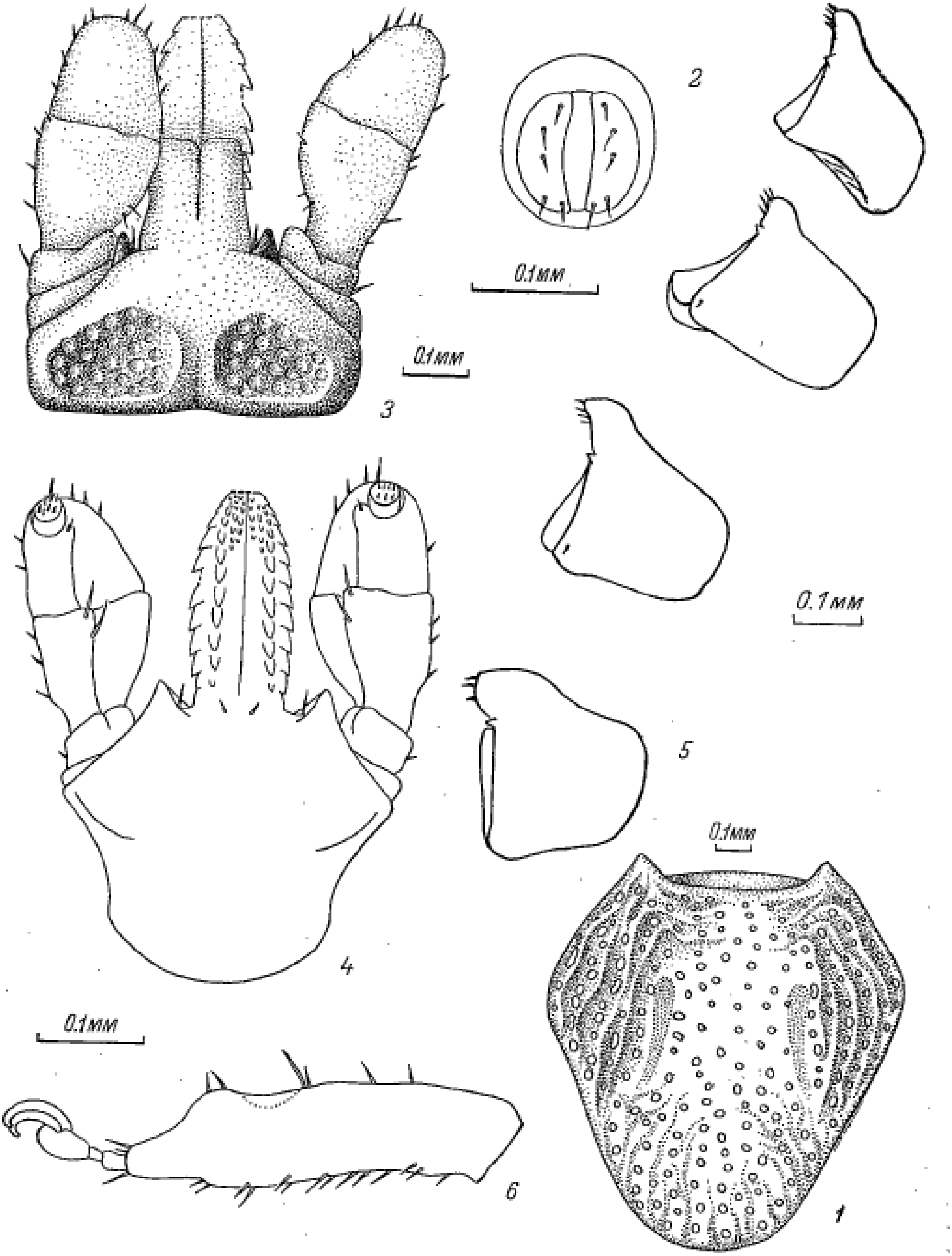

0,1мм
0,1мм
0,1мм
0,1мм
0,1мм
1
2
3
4
5
6
