# Supplementary figures and images for: Phylogenetic analyses of Ixodes rugicollis with notes on its morphology in comparison with Ixodes cornutus
Source: Parasit Vectors. 2023 Mar 16;16:106. doi: 10.1186/s13071-023-05718-z (PMC10022209; doi:10.1186/s13071-023-05718-z)

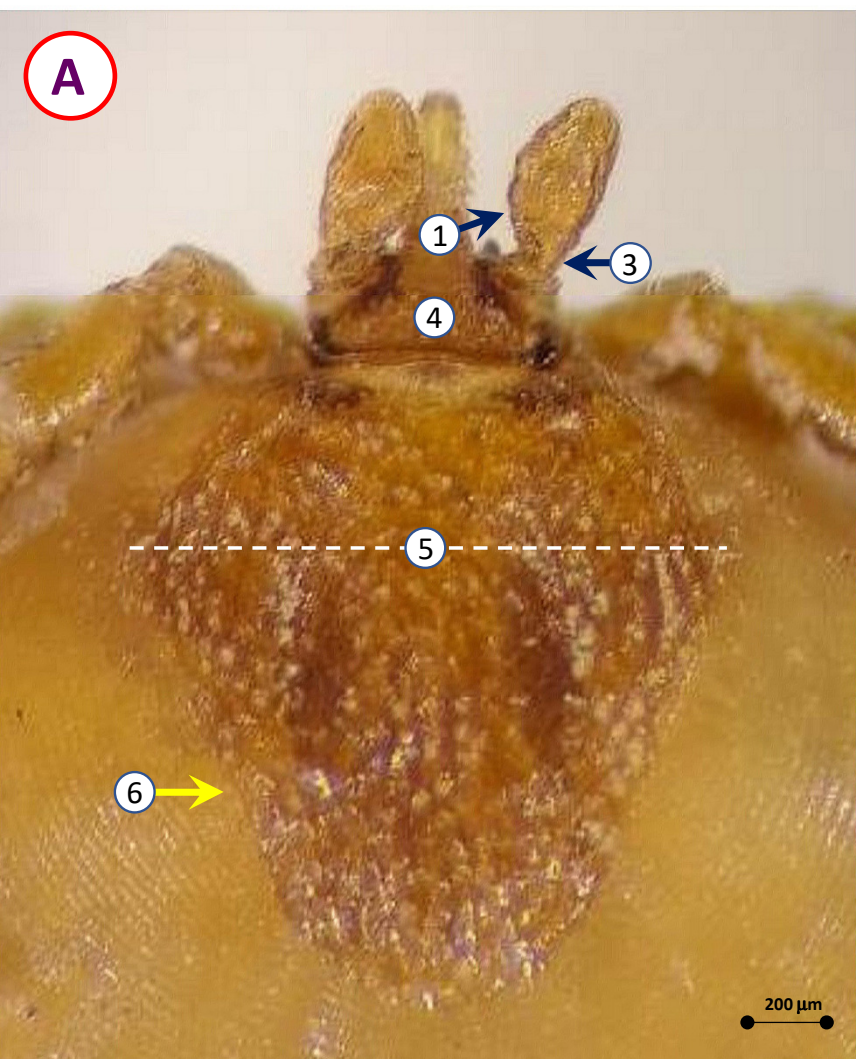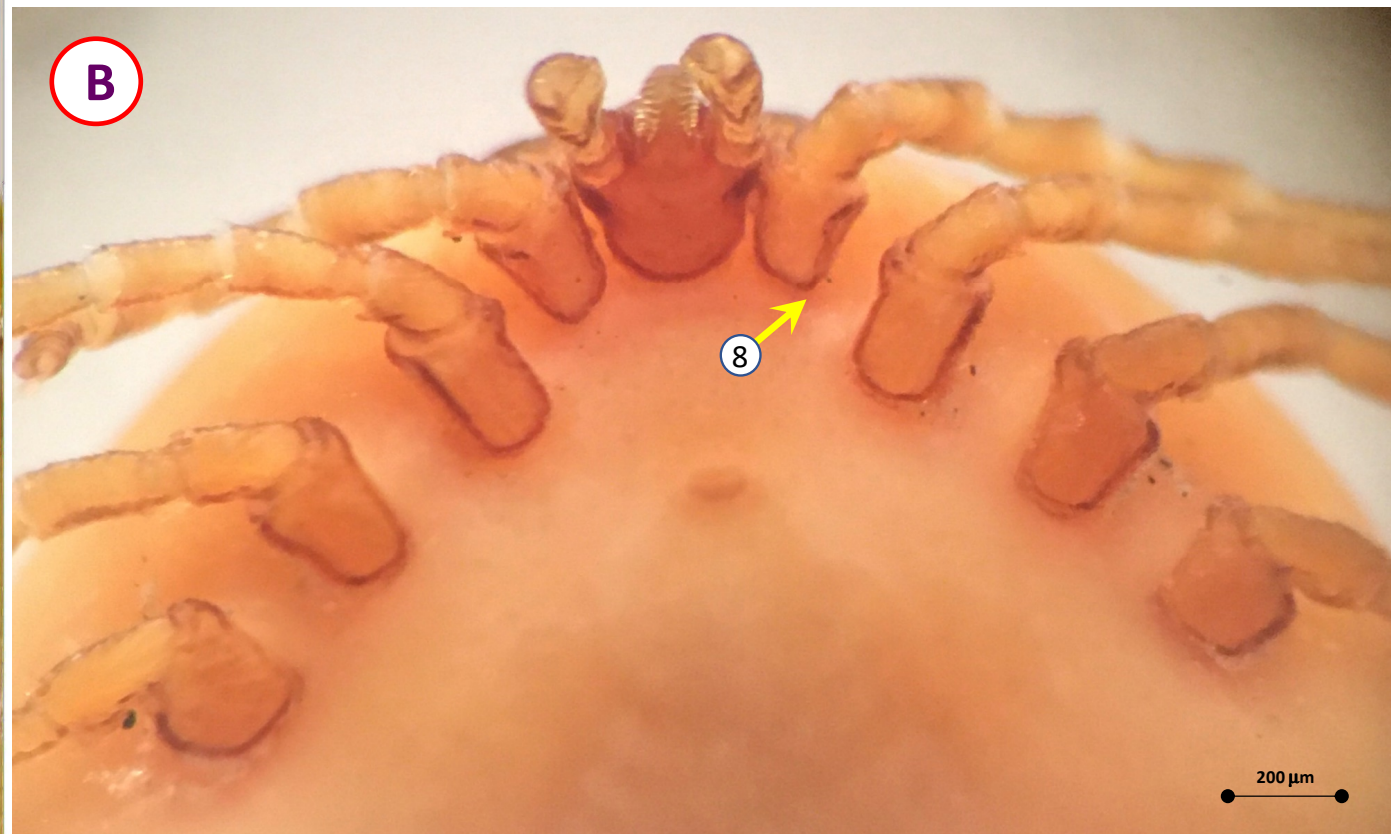

Supplement: Supplementary file 2 — Additional file 2: Low-resolution pictures of Ixodes cornutus lectotype (female) stored at ZIN-RAS (St. Petersburg, Russia): (A) dorsal view of scutum and basis capituli in dry mount (dashed line marks maximum width of the scutum); (B) ventral view of anterior idiosoma in wet mount. Numbers between 1 and 8 mark structures of diagnostic importance described in Table 1. [file 13071_2023_5718_MOESM2_ESM.pdf]

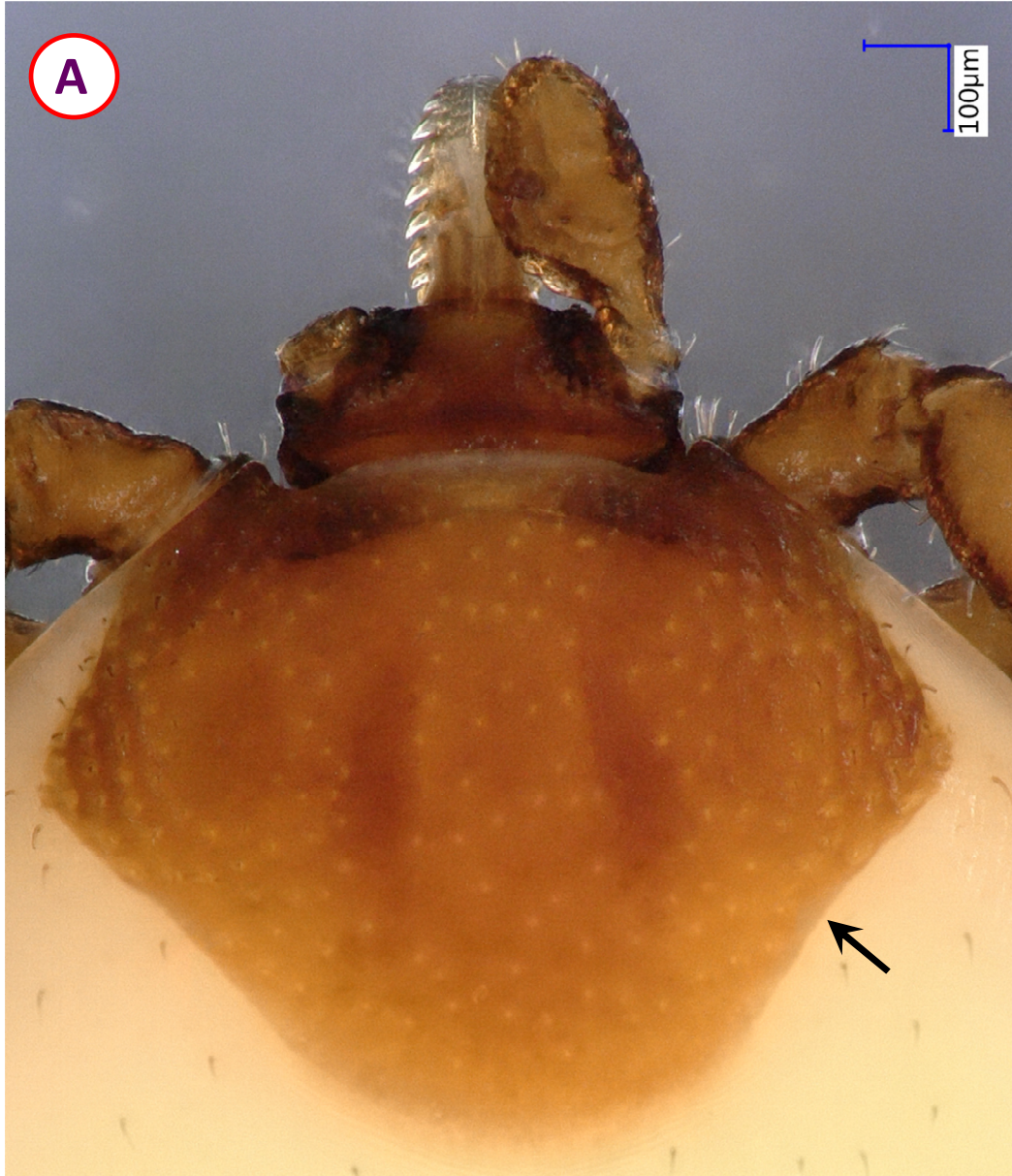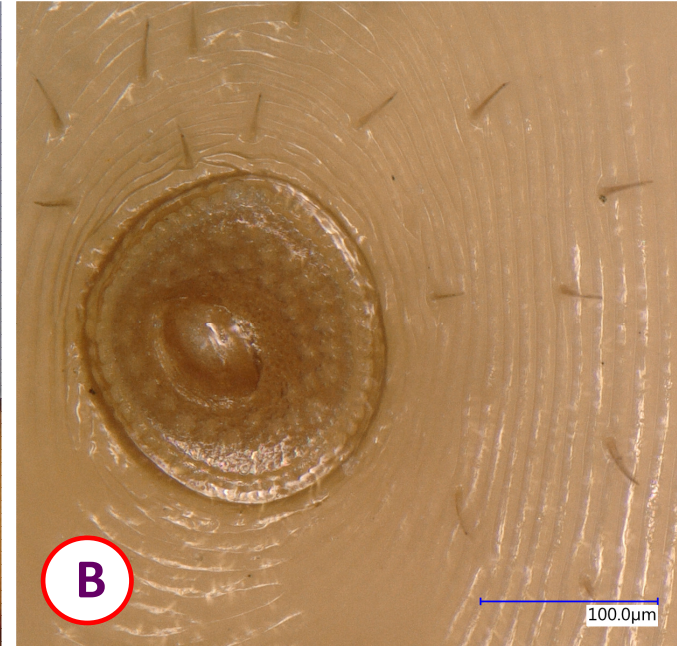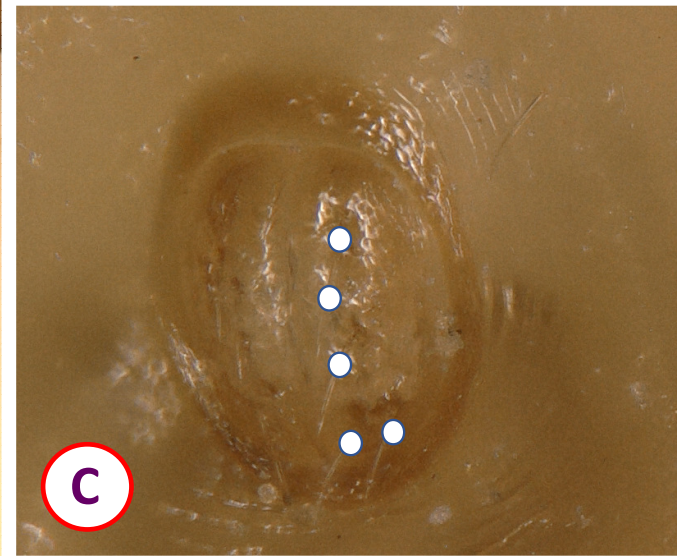

Supplement: Supplementary file 3 — Additional file 3: Morphological characters of Ixodes rugicollis female: (A) scutum and basis capituli in wet mount shown in a slightly posterior view (the dark arrow indicates concave posterolateral margin of the scutum); (B) respiratory opening and (C) anal valves with five pairs of hair (marginated white circles indicate the base of these hairs to highlight their arrangement). [file 13071_2023_5718_MOESM3_ESM.pdf]

A

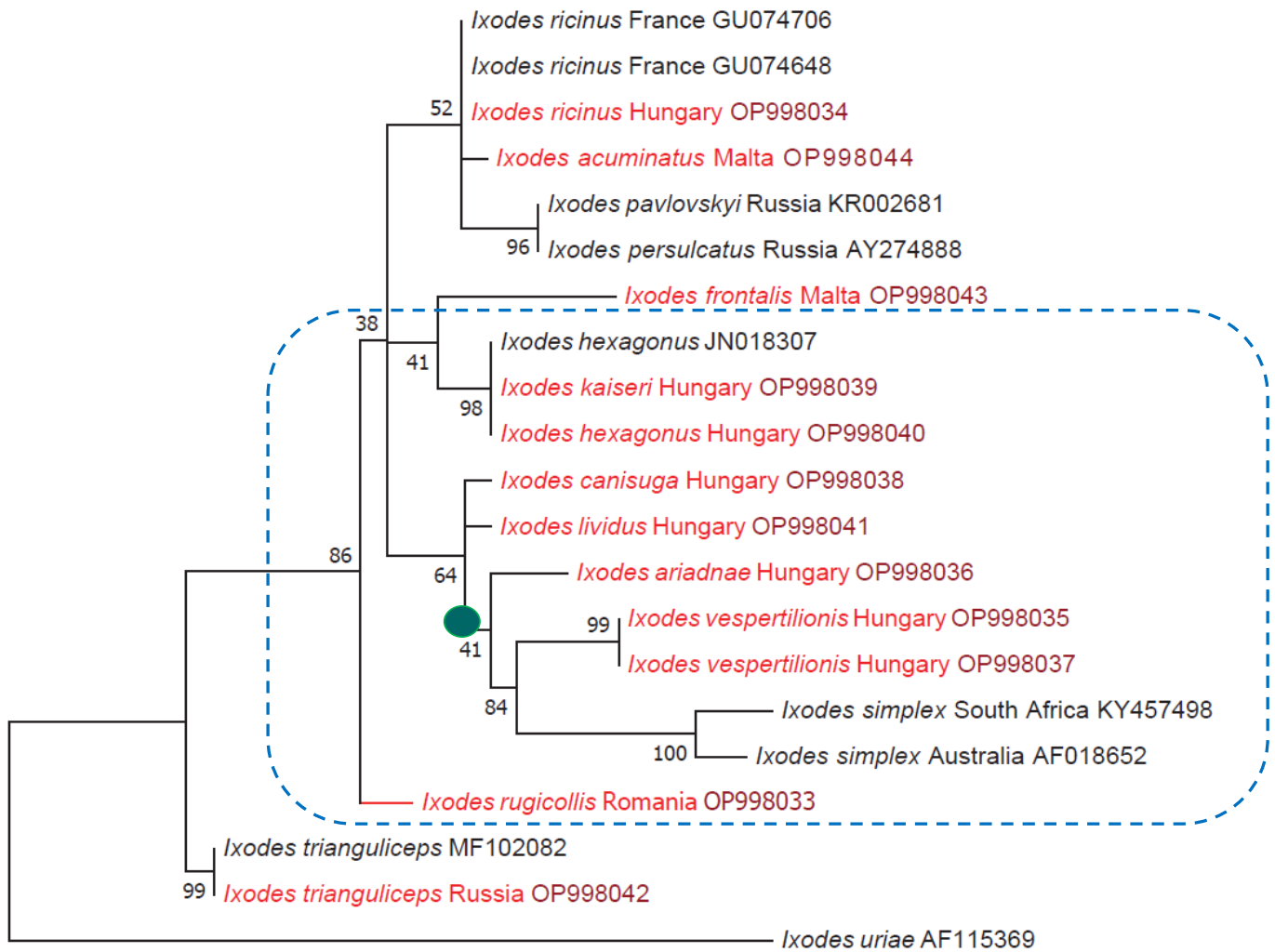

0.005

B

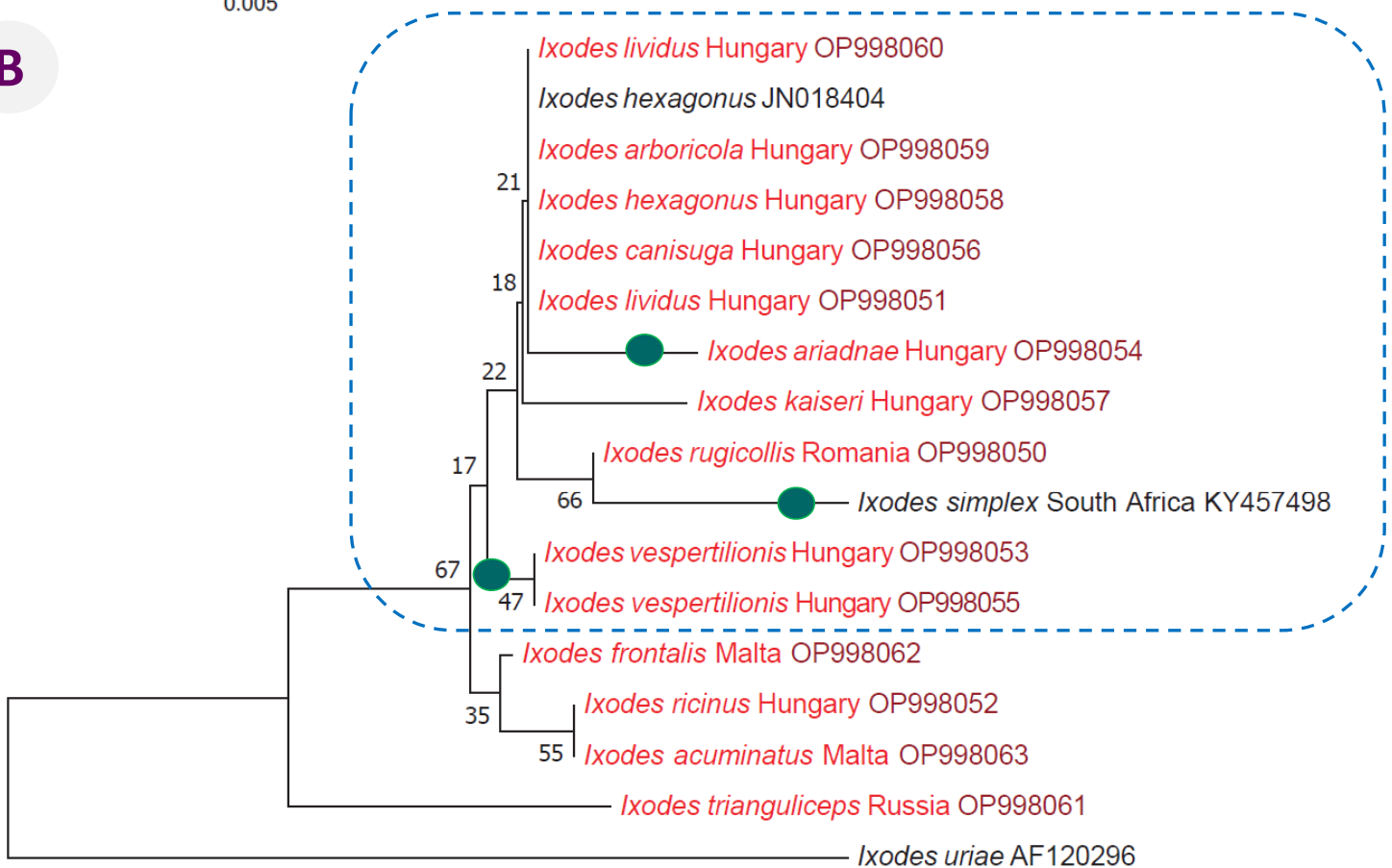

0.0020

Supplement: Supplementary file 5 — Additional file 5: Phylogenetic tree of ixodid ticks based on (A) 18S and (B) 28S rRNA gene sequences. In each row of individual sequences, the country of origin and the GenBank accession number are shown after the species name. Ixodes rugicollis is marked with red branch (A) and all sequences from this study with red fonts and maroon accession numbers. The subgenus Pholeoixodes is surrounded by a blue dashed line, and Eschatocephalus species are marked with a green dot on the branch. The evolutionary history was inferred by using the (A) maximum likelihood method based on the Jukes-Cantor model or (B) neighbor-joining method and p-distance model. The tree with the highest log likelihood is shown. The percentage of trees in which the associated taxa clustered together is shown next to the branches. The tree is drawn to scale, with branch lengths measured in the number of substitutions per site. The analysis involved 21 and 17 nucleotide sequences for the 18S and 28S rRNA genes, and there were a total of 1023 and 586 positions in the final dataset, respectively. All positions containing gaps and missing data were eliminated. [file 13071_2023_5718_MOESM5_ESM.pdf]
