# Supplementary material for: Phylogenetic analyses of Ixodes rugicollis with notes on its morphology in comparison with Ixodes cornutus
Source: Parasit Vectors. 2023 Mar 16;16:106. doi: 10.1186/s13071-023-05718-z (PMC10022209; doi:10.1186/s13071-023-05718-z)

Additional File 4. Selected measurements of females of *Ixodes rugicollis* from Romania and France, in comparison with the lectotype of *Ixodes cornutus* from Tajikistan. The structures that were measured are illustrated below the table, based on the drawing of *I. cornutus* from Filippova (1977). Measurements are provided in mm.

Structure	Parameter	Country of origin		
		Romania	France	Tajikistan
**(1) Scutum**	**Length**	1.02	0.98	1.26
	**Width**	1.09	-*	1.19
	**Index**	0.94	-	1.06
**(2) Basis capituli**	**Length**	0.25	0.24	0.31
	**Width**	0.48	0.44	0.56
	**Index**	0.52	0.55	0.55
**(3) Anterior, broad part of palps**	**Length**	0.31	0.38	0.4
	**Width**	0.19	0.22	0.22
	**Index**	1.63	1.73	1.8

* The scutum of this specimen is asymmetrical, therefore it was excluded form width and index comparison

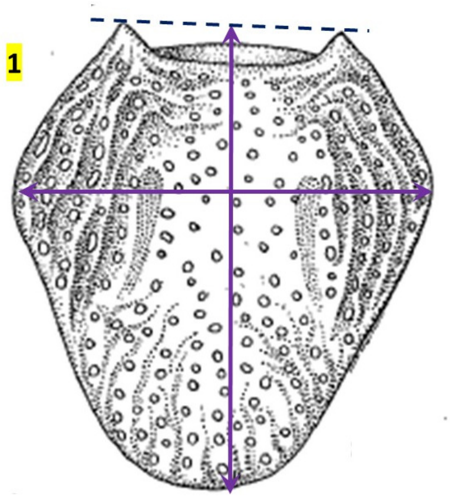

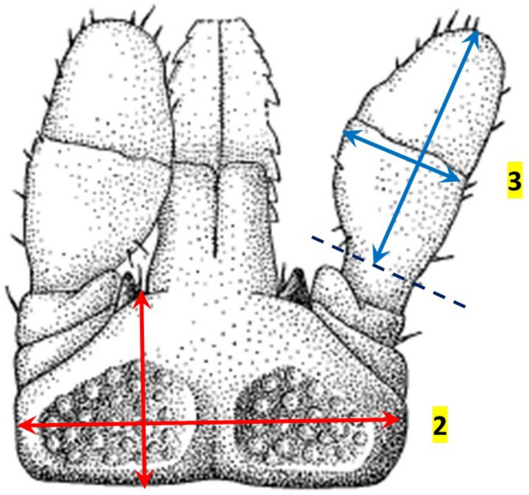

Supplement: Supplementary file 4 — Additional file 4: Measurements of Ixodes rugicollis and I. cornutus. [file 13071_2023_5718_MOESM4_ESM.pdf]
